# The High Dispersion of Phenolic Concentration in the Seeds, Skins and Wines of *Vitis vinifera* L. Cultivars Limits Comparative Analysis and Varietal Categorization Under Heterogeneous Conditions

**DOI:** 10.3390/antiox15101355

**Published:** 2026-10-20

**Authors:** Remigio López-Solís, Eliana Seguel-Rubio, Claudio Muñoz-Pérez, Mónica Vergara-López, Rocío Belmar-Díaz, Marcela Medel-Marabolí, Danilo Aros-Orellana, Jaime Laqui-Estaña, Elías Obreque-Slier

**Affiliations:** 1 Interdisciplinary Group of Cell Biology and Genetics, Faculty of Medicine–ICBM, University of Chile, Independencia 1027, Santiago 8380453, Chile; rlopez@med.uchile.cl; 2 Department of Agro–Industry and Enology, Faculty of Agronomical Sciences, University of Chile, Santa Rosa 11315, Santiago 8820808, Chile; eliseguel@gmail.com (E.S.-R.); claudio.munoz.perez@ug.uchile.cl (C.M.-P.); rociobelmardiaz@gmail.com (R.B.-D.); mmedel@uchile.cl (M.M.-M.); daros@uchile.cl (D.A.-O.); 3 School of Technical Education, Faculty of Agricultural Sciences, University of Talca, Ramón Sanfurgo 980, Santa Cruz 3130000, Chile; movergara@utalca.cl; 4 Sensory Analysis and Consumer Study Group, Escuela Profesional de Ingeniería Agroindustrial, Universidad Nacional de Moquegua, Prolongación Calle Ancash s/n, Moquegua 18001, Peru; j272329@dac.unicamp.br; 5 Department of Food Science and Nutrition, School of Food Engineering, University of Campinas, Monteiro Lobato Street 80, Campinas 13083-862, São Paulo, Brazil

**Keywords:** tannin, anthocyanin, flavanol, grape seed, grape skin

## Abstract

This comparative literature review evaluated the polyphenolic profiles in seeds, skins, and wines of nine *Vitis vinifera* L. cultivars. The data compilation revealed that total tannins and catechin exhibited comparatively more homogeneous phenolic profiles in seeds than skins, suggesting differential tissue regulation of specific phenolic biosynthetic pathways within the berry. However, this pattern did not extend to total phenols or epicatechin. Furthermore, the results confirmed wide inter- and intra-cultivar polyphenolic variations, with intra-cultivar dispersion frequently reaching a magnitude comparable to, or exceeding, that observed between cultivars. These variations are likely related to the multifactorial effect of environmental, genetic, and cultural contexts on polyphenol concentration in grapes and wines due to methodological differences (extraction protocols, various quantification techniques and winemaking practices) and biological factors (edaphic and climatic conditions). Furthermore, this high inter- and intra-cultivar variability leads to overlapping concentration ranges, preventing strict varietal categorization. This demonstrates that each study represents only a snapshot of specific environmental and agricultural conditions. Consequently, this review underscores that standardized analytical protocols, rigorous experimental designs, and robust biological replication are essential to distinguish between experimental noise and biological variability, reinforcing the value of comprehensive phenolic profiles for genotypic characterization and targeted winemaking decisions.

## 1. Introduction

Phenolic compounds are a group of secondary metabolites derived from plants that influence the chemical characteristics of *Vitis vinifera* grapes and have consequently been linked to wine quality [1,2]. The polyphenols present in the skins, seeds and stems (and even in the pulp of some red grape varieties) are extracted during the winemaking process [3,4] and contribute to various sensory characteristics of wines, such as color, aroma, astringency, and bitterness [1,3,5,6]. Furthermore, phenolic compounds are classified into two major groups: non-flavonoids (phenolic acids, cinnamic acids and stilbenes) and flavonoids (flavonols, anthocyanins, and flavanols or proanthocyanidins) [3,5]. Anthocyanins are the compounds responsible for the color of skins and wines, while flavanols are associated with astringency and bitterness. Finally, flavonols are thought to contribute to the color and bitterness of wines. Beyond their sensory relevance, these compounds are also widely recognized as major contributors to the antioxidant capacity of grapes and wine, a property inherent to their chemical structure, particularly the hydroxyl groups on the aromatic rings, which confer high free-radical scavenging and reducing capacity [1,2,3,4,5,6,7,8,9,10].

Various reports have highlighted the numerous factors that modulate the development and availability of polyphenols in grape skins, seeds, and wines [3,7,8,9,10]. A factor frequently reported in the literature links the effect of wine grape variety to the concentration of these polyphenols in grape tissues and, consequently, in wine [11,12,13,14]. Numerous studies have been designed under strict experimental and methodological conditions to comparatively evaluate the varietal factor, thereby controlling for other countless factors that could influence the concentration of this group of metabolites. Despite the above, comparative phenolic analysis of *Vitis vinifera* grapes and wines is not free of challenges, since, as previously noted, these vegetal metabolites are quantitatively modulated by multiple environmental and technological factors. This particular condition means that any variation in the experimental design affects the availability of these analytes and, consequently, makes it difficult to compare the phenolic profiles of the same variety grown at different growing latitudes and/or under various environmental and technological conditions. For this reason, this study proposes a comparative analysis of the phenolic composition in seeds, skins, and wines from nine *Vitis vinifera* L. varieties to determine the phenolic variability exhibited by these cultivars under broadly diverse conditions, which would consequently contribute to a better understanding of the behavior of these important secondary metabolites. Unlike previous reviews on grape and wine phenolics, this study integrates seed, skin, and wine data within the same analytical framework across nine internationally relevant cultivars, enabling direct cross-matrix comparisons that are rarely addressed jointly in the existing literature.

## 2. Materials and Methods

### 2.1. Literature Search and Study Selection

This study utilized various bibliographic resources obtained from Web of Science and Scopus (2005–2025). The search keywords were “phenolic,” “polyphenols,” “grape seeds,” “grape skins,” and “wines” combined with each cultivar name. For cultivars with recognized international synonyms, both the primary name and its synonym were used as search terms: ‘Mourvèdre’ OR ‘Monastrell’, ‘Syrah’ OR ‘Shiraz’, and ‘Grenache’ OR ‘Garnacha’. No additional synonyms were searched for the remaining cultivars (Cabernet Sauvignon, Carménère, Malbec, Merlot, Sangiovese, and Tannat). Retrieved records were screened by title and abstract to identify studies reporting quantitative phenolic data (mean and standard deviation, or equivalent) for at least one of the three plant matrices (seeds, skins, or wine) in each of the nine cultivars. Claude Haiku 5.5 was used by some co-authors to help distinguish, among the retrieved scientific records, those that reported quantitative phenolic data for at least one of the three plant matrices. The AI-assisted screening results were independently verified by the authors, and the final decisions regarding study inclusion and data extraction were made by the authors. Given the exploratory, comparative nature of this narrative review, database sources and inclusion/exclusion criteria described above are reported in full to support transparency and reproducibility. The methodology involved an initial stage of gathering information relevant to the stated objective. Next, studies were selected that had exclusively examined the phenolic content of the cultivars’ seeds, skins, and wines. The selection of the nine cultivars included in this study, relative to a broader initial pool of candidate varieties considered during the literature search, was based on two combined criteria: (i) their recognized relevance and wide geographical distribution across major wine-producing countries, and (ii) the availability of a sufficient number of studies reporting quantitative phenolic data across all three plant matrices (seeds, skins, and wines) for the same cultivar. Other internationally relevant varieties were excluded from the final analysis due to an insufficient number of studies reporting data across all three matrices simultaneously, which would have precluded a balanced cross-matrix comparison for these cultivars. The final set of included studies was restricted to sources indexed in Web of Science and/or Scopus. Unpublished theses and other non-indexed grey literature were excluded from the final dataset to ensure methodological consistency with the stated search strategy.

### 2.2. Data Extraction

After conducting a thorough review of the available sources of information, the material was selected and classified. Quantitative data were obtained from articles that reported the mean ± standard deviation. Prior to compilation, all reported values were normalized to a common expression basis (fresh weight, whole-berry equivalent) and reference standard for each compound class to ensure comparability of the concentration ranges across studies employing different original units and calibration standards. Studies that did not report sufficient information to allow this conversion (e.g., lacking mean berry or seed weight, moisture content, or a clearly specified reference standard) were excluded from the final dataset; consequently, all values retained in the compiled dataset (Tables S1–S3, Supplementary Material) are expressed on a common, directly comparable basis. For articles that presented different treatments for the same variety and reported more than one mean ± standard deviation, the minimum and maximum values reported across the treatments were considered. In articles that reported only one mean ± standard deviation, the minimum value (mean − standard deviation) and the maximum value (mean + standard deviation) were calculated.

### 2.3. Cultivars

Data analysis and discussion were conducted on the phenolic composition (general and detailed) of nine *Vitis vinifera* L. varieties included in the study: Cabernet Sauvignon (CS), Merlot (ME), Carménère (CA), Syrah (SY), Malbec (MB), Sangiovese (SG), Tannat (TA), Mourvèdre (MV), and Grenache (GA). The selection of these nine cultivars, relative to a broader initial pool of candidate varieties considered during the literature search, was based on two combined criteria: (i) their recognized relevance and wide geographical distribution across major wine-producing countries, and (ii) the availability of a sufficient number of studies reporting quantitative phenolic data across all three plant matrices (seeds, skins, and wines) for the same cultivar. Other internationally relevant varieties were excluded from the final analysis due to an insufficient number of studies reporting data across all three matrices simultaneously, which would have precluded a balanced cross-matrix comparison for these cultivars. Finally, Mendeley software (version 1.19.8) was used to organize bibliographic references.

### 2.4. Statistical and Graphical Analysis

Box plots were constructed based on the individual data collected from the literature review for each cultivar (Figure 1, Figure 2 and Figure 3). Each box represents the interquartile range (IQR), spanning from the first quartile (Q1) to the third quartile (Q3), which contains the central 50% of the data. The inner horizontal line represents the median, which was used as a measure of central tendency due to the asymmetry of the data. Potential outliers were identified using Tukey’s criterion, with the lower and upper limits of the whiskers set at Q1 − 1.5 × IQR and Q3 + 1.5 × IQR, respectively. All analyses were performed using R software version 2026.05.0. To complement the graphical dispersion analysis, the coefficient of variation (CV) was calculated for each cultivar–compound combination as CV (%) = (standard deviation/mean) × 100, using the same set of individual compiled values from the literature underlying the box plots (Table S4, Supplementary Materials). Because these values originate from independent studies employing heterogeneous analytical protocols, extraction methods, and normalization procedures, the resulting CV reflects the combined contribution of true biological variability and inter-study methodological variability, and should be interpreted as a descriptive measure of overall dispersion rather than a precise estimate of intrinsic biological variance.

## 3. Results and Discussion

This study conducted a comparative literature review of the concentrations of various phenolic compounds present in seeds, skins, and wines. To this end, we reviewed research conducted on nine wine grape varieties, which were selected for their iconic status and/or high relevance to the wine industry in wine-producing countries, such as France (Merlot), Spain (Grenache and Mourvèdre), Australia (Syrah), Argentina (Malbec), Italy (Sangiovese), Uruguay (Tannat), and Chile (Carménère). Cabernet Sauvignon, meanwhile, has traditionally been considered one of the most widely cultivated varieties in the world. Tables S1–S3 show (Supplementary Material) the content of the various phenolic compounds reported in seeds, skins, and wines from the different varieties considered in this study. Each Table indicates the phenolic parameter studied and the minimum and maximum levels reported by the various studies. Concurrently, the individual concentrations reported in different studies were plotted as box plots as previously described (Figure 1, Figure 2 and Figure 3). Four central ideas emerged from this analytical study.

### 3.1. Polyphenol Biosynthesis Undergoes Compound-Specific Tissue Regulation

An initial observation derived from this study showed that compounds associated with condensed tannins exhibited comparatively more homogeneous profiles in seeds than in skins—total tannins (Coefficient of variation = 101.6% in seeds versus 125.2% in skins) and catechin (Coefficient of variation = 97.4% versus 124.9%)—consistent with a biosynthesis confined to the early stages of berry development (Table S4, Supplementary Material). However, this pattern was not observed for other compounds: total phenols showed slightly greater dispersion in seeds than in skins (Coefficient of variation = 65.9% versus 58.6%), as did epicatechin (Coefficient of variation = 101.9% versus 76.3%), while total anthocyanins could not be evaluated in seeds given their trace or absence in this tissue (Table S4, Supplementary Material). This suggests that tissue regulation of phenolic biosynthesis is not a uniform phenomenon across all polyphenol classes, but rather depends on the specific biosynthetic pathway and its activation timing during berry development, and that the biosynthesis and accumulation of these specific compounds may be subject to differential tissue regulation in the berry.

This phenomenon could be attributed to three non-mutually exclusive explanations: (1) The seed is an embryonic tissue whose phenolic composition may be primarily determined by genetically controlled developmental dynamics and less subject to the direct influence of external climatic factors. Conversely, the berry skin is in direct contact with the environment, serving as the main protective interface against solar radiation, ambient temperature, humidity, and fungal diseases, among other factors [15,16,17]. (2) The synthesis of condensed tannins in seeds occurs predominantly during the early stages of berry development and ceases prior to veraison [18,19], whereas phenolic synthesis in skins is modulated during late ripening [18,20,21,22]. (3) Genetic variability may exert a less pronounced phenotypic expression in seed phenolic biosynthesis if this pathway is governed by regulatory mechanisms that are more evolutionarily conserved. It should be noted that the number of independent studies (*n*) contributing to each cultivar–compound estimate ranged from 1 to 11, and CV estimates based on a low number of studies (*n* ≤ 2) (Table S4, Supplementary Materials) should be interpreted with appropriate caution, as they are less robust indicators of true biological variability than those derived from larger study counts. More broadly, given that the compiled values originate from studies with heterogeneous methodologies (Section 2.4), the reported CVs should not be interpreted as precise measures of intrinsic biological variance, but rather as descriptive indicators of the overall dispersion observed across the compiled literature.

### 3.2. Grape Variety Influences Polyphenol Accumulation

A second observation supported by this study reinforces the traditional notion of “inter-cultivar variability” in the concentration of polyphenols in *Vitis vinifera* grapes and wines. For example, the Syrah variety stood out for having the highest average concentration of total phenols and gallic acid in seeds. Similarly, the Malbec variety had the highest average catechin and epicatechin content in seeds, while the Mourvèdre variety stood out for having the highest concentrations of total phenols, catechin, and epicatechin in skins. Taken together, these results for Syrah, Malbec, and Mourvèdre consistently position them as the genotypes reporting the highest phenolic values across multiple compounds in seeds and skins. This convergence across compounds, rather than an isolated pattern in a single parameter, suggests (though does not conclusively establish, given the methodological heterogeneity among the compiled studies) that grape variety may be an influential factor in polyphenolic accumulation in skins and seeds. Confirming this pattern as a genuine varietal characteristic, rather than an artifact of unevenly distributed methodological or environmental conditions across studies, would require standardized comparative trials under controlled growing and analytical conditions [10,14,23].

### 3.3. Polyphenol Concentrations Vary Widely Within Single Cultivars

A third observation resulting from this study would demonstrate the existence of a very wide “intra-cultivar” variation in polyphenol content in *Vitis vinifera* grapes and wines. For instance, Syrah seeds displayed a particularly broad dispersion in total phenols, with values ranging from 27.7 mg/g to values exceeding 600 mg/g within the same cultivar. This generated a considerably wider interquartile range than those observed in Cabernet Sauvignon or Merlot, which exhibited much more homogenous profiles. This example illustrates a broader pattern observed throughout this study: in several cultivar–compound combinations, the dispersion of values reported within a single cultivar was comparable to, or even exceeded, the differences observed between distinct cultivars, challenging the assumption that inter-cultivar comparison alone can reliably capture the true phenolic identity of a variety. A similar pattern was observed in the total tannin content of Merlot skins, where individual replicates were widely distributed between approximately 0.1 and 95.9 mg/g. In the case of Sangiovese wines, the most notable dispersion was recorded in phenolic acids, with a range of values spanning concentrations between 14.7 and 361.9 mg/L. This high phenolic dispersion in the same variety, which is represented in this study with several examples, would have its origin in the impact of multiple environmental and technological factors on the biosynthesis of these secondary metabolites [5,20,23,24,25]. Despite the high intra-cultivar variability observed in several phenolic compounds, one aspect that deserves particular attention beyond direct comparisons between cultivars is the recurrence of outliers observed in different tissues and cultivars throughout this study, for example, in Syrah seeds (total tannins, catechin and epicatechin) and also in the skins of Grenache, Syrah and Tannat (total anthocyanins, catechin and epicatechin). These and other exceptions appear to correspond to isolated events in specific samples, which could reflect a greater susceptibility of certain cultivars to specific management, environmental, or even clonal factors, rather than an intrinsically distinct tissue regulation. Furthermore, this pattern (where a cultivar presents individual replicates with extraordinarily high concentrations compared to samples from different studies) raises a relevant methodological/biological question that transcends the analysis of any single tissue. In any case, the distinction between these two possibilities (methodological or biological) cannot be resolved with the current study design; however, their identification is valuable for future research. Subsequent studies could benefit from a larger number of biological replicates per cultivar, as well as a more detailed record of vineyard environmental conditions, in order to discriminate with greater certainty between experimental variability and true biological variation.

### 3.4. Multifactorial Contexts Restrict Varietal Comparison and Categorization

The three observational proposals described above together generate a highly relevant challenge that is the basis of this study: the multifactorial effect of environmental, genetic, and cultural contexts on polyphenolic concentrations in grapes and wines restricts the comparative analysis and categorization of distinct *Vitis vinifera* L. varieties. As previously mentioned, the concentration of polyphenols in seeds, skins, and wine is highly dependent on a wide range of factors, which can be classified into methodological and biological variables. From a methodological perspective, the analytical techniques used to determine these chemical compounds are highly diverse. In principle, these techniques include different analytical procedures for extracting these analytes from seeds and skins under varying operational conditions. Furthermore, there is a wide array of methodological proposals for the analysis and quantification itself. For instance, regarding the quantification of total phenols, two methodologies are widely used (absorbance at 280 nm and the Folin–Ciocalteu method [26]), whereas total tannin content is preferentially determined using the techniques proposed by Bate-Smith, Porter et al. and Mercurio et al. [27,28,29]. In the case of total anthocyanins, several other techniques are also frequently reported [30,31,32,33]. Likewise, the methodologies for detailed phenolic profiling are even more diverse. For example, for the determination of anthocyanin profiles, the proposals described by González-San José et al. [34], Carbonneau and Champagnol [35], Revilla et al. [36], Monagas et al. [37], Chira et al. [38], García-Marino et al. [39], Giuffré [40], Gori et al. [41], Gil et al. [42] and Gordillo et al. [43], among others, are available. Similarly, for the quantification of low-molecular-weight phenols, various numerous methodological approaches have been published [11,44,45,46,47,48,49,50,51,52,53,54,55]. Furthermore, in addition to spectrophotometric approaches, several of the compiled studies used chromatographic and mass spectrometry-based techniques (such as HPLC-DAD, LC-MS/MS, and UHPLC) for the detailed profiling of individual phenolic compounds, which differ substantially from spectrophotometric methods in sensitivity, selectivity, and quantification limits. Beyond the mere coexistence of these methodological approaches, it is relevant to briefly consider their comparative specificity and potential contribution to the observed inter-study variability. Spectrophotometric assays (e.g., Folin–Ciocalteu, absorbance at 280 nm) are rapid and widely accessible, but are inherently less specific: their absorbance-based readouts can be influenced by co-extracted non-phenolic compounds, potentially inflating reported concentrations in a manner that varies with sample matrix and extraction protocol. In contrast, chromatographic and mass spectrometry-based techniques allow the separation and identification of individual phenolic compounds, offering substantially higher specificity and lower susceptibility to matrix interference, but often at the cost of higher analytical complexity and cost, and with results that remain dependent on the reference standards and calibration curves. Consequently, part of the inter-study variability reported in this review is likely attributable not only to genuine biological or environmental differences, but also to the differential specificity and susceptibility to interference inherent to the analytical method employed in each study. A systematic comparison of the analytical performance of each technique is beyond the scope of this review. However, a dedicated comparative analysis of analytical methodologies applied to grape and wine phenolics—evaluating their relative sensitivity, reproducibility, and comparability of results—constitutes a valuable direction for future research. The availability of a very wide variety of methodologies is a relevant factor for research studying any variety. Nevertheless, the use of methodological protocols that employ different solvents, times, temperatures, and other experimental conditions hinders contrasting comparative analyses. In any case, the high intra- and inter-cultivar dispersion reported in this study, which in several cases was of comparable or greater magnitude within a single cultivar than between different cultivars, reinforces the need for additional analytical verification either by evaluating potential cross-contamination or errors during analysis. Accordingly, in these cases, reanalysis of samples or use of a complementary analytical technique would be advisable before accepting the observed values as part of the natural biological variability of the cultivar. From a biological perspective, it cannot be ruled out that intra- and inter-cultivar phenolic differences reflect genuine phenomena of heterogeneity within and between cultivars. Nonetheless, mounting experimental evidence supports the significant impact on the synthesis of polyphenols displayed by diverse soil–climate and winemaking conditions, which can be grouped into edaphoclimatic conditions, enological–viticultural management practices, and grape variety [5,56,57]. In grapevine, it has been reported that soil nutrition [58,59,60,61,62,63], vineyard vigor [64], soil characteristics and water deficit [60,61,64,65,66,67], hormone levels [68,69,70,71,72,73,74], the presence of wounds and diseases [75,76], vineyard altitude [77,78], light intensity [66,79,80,81,82,83], temperature [66,84,85,86,87], and humidity [88,89] are crucial factors in the synthesis of polyphenols in berry seeds and skins. In addition, various factors impact the phenolic composition of wines, such as fermentation temperature, pressing intensity, pump-over frequency, maceration time supplementation with commercial tannins [90,91,92], barrel aging [93], wood format [94], oak species [95], wood toasting level [96,97,98], barrel age [99,100], aging time [101], and the specific wine variety destined for aging [94], among others. Furthermore, the present study revealed that the behavior of the polyphenolic matrix of one cultivar is not necessarily the same in another, because the transfer of polyphenols from skins and/or seeds to the wine is neither selective nor linear across cultivars. For example, Tannat and Grenache have the highest average values of total tannins in seeds and skins, but Malbec exhibits this same atypical pattern in wines. Similarly, Mourvèdre and Syrah show the highest concentrations of total phenols in seeds and skins, respectively, but Cabernet Sauvignon wine has the highest average values of these chemical compounds. This lack of direct correlation between matrices suggests that all aspects considered during polyphenol extraction during vinification decisively modulate which compounds ultimately predominate in the wine, regardless of their abundance in the raw material. In any case, differentiating between these perspectives (methodological or biological) is beyond the scope of this study, but it represents a relevant direction for future research, ideally incorporating stratified sampling by methodological and biological variables.

## 4. Future Projections on the Topic

These findings represent a step beyond simply confirming that environmental and methodological factors influence phenolic variability by providing a cross-matrix, multi-cultivar quantitative framework that demonstrates the practical limits of using phenolic content as a varietal descriptor, and by identifying which specific phenolic classes are more or less susceptible to this variability. We acknowledge as a limitation of this review that a systematic documentation of individual study characteristics (e.g., sample size, growing region, vintage, and analytical technique) was beyond its current scope. Compiling and cross-referencing this metadata across the full set of studies would represent a valuable extension for future, more granular syntheses of grape and wine phenolic variability. Finally, based on the methodological heterogeneity identified throughout this review, we propose the following minimum reporting checklist to improve the comparability of future studies on grape and wine phenolic composition: (a) analytical method and reference standard used for each compound class; (b) expression basis of reported values (for example fresh versus dry weight, per-berry, etc.); (c) sample size (number of biological replicates per treatment/condition); (d) growing season in which samples were collected; (e) geographic origin and growing conditions; and (f) cultivar clone or accession and, when known, given potential intra-varietal genetic variability. Adoption of these minimum reporting elements would enable future comparative reviews to move beyond descriptive range compilation toward formal variance-partitioning analyses (e.g., mixed-effects models) capable of quantifying the relative contribution of varietal, environmental, and methodological sources to the observed phenolic variability.

## 5. Conclusions

In brief, the aforementioned background suggests that the high intra- and inter-varietal phenolic variability—with intra-cultivar variability frequently matching or surpassing inter-cultivar differences—creates a critical situation when comparative analyses are performed between wine grape varieties, since even minimal modifications to any viticultural and/or oenological condition can impact phenolic concentration. Thus, this multiplicity of factors involved in the synthesis of these metabolites would allow the generation of different phenolic matrices in grapes and wines, which would further contribute to the comparative problem of the same variety cultivated in different latitudes or under different oenological management. Furthermore, it is highly probable that subtractive, additive, and/or synergistic relationships (scarcely studied) exist among the various factors, potentially leading to highly specific phenolic expression and representing a significant derivative that would further exacerbate the problem addressed in this study. In parallel, the results of this study reveal a certain difficulty in classifying grape varieties according to their phenolic content, since the wide dispersion of these chemical compounds in grapes and wine generates a clear overlap in concentrations among varieties, preventing strict categorization. In summary, each research proposal would be a “snapshot” of a particular moment with specific environmental–agricultural conditions and should in no case be equated with other experimental conditions. Therefore, comparative studies that employ experimental designs with strict standardization of growing area conditions and/or winemaking practices to study one or more factors would contribute to clarifying the accumulation dynamics of these secondary metabolites, so relevant to wine production. Furthermore, phenolic quantification in different tissues of the grape cluster and wine reinforces the value of phenolic profiles as a tool for genotypic characterization and as an objective criterion for guiding winemaking decisions.

## Figures and Tables

**Figure 1:**
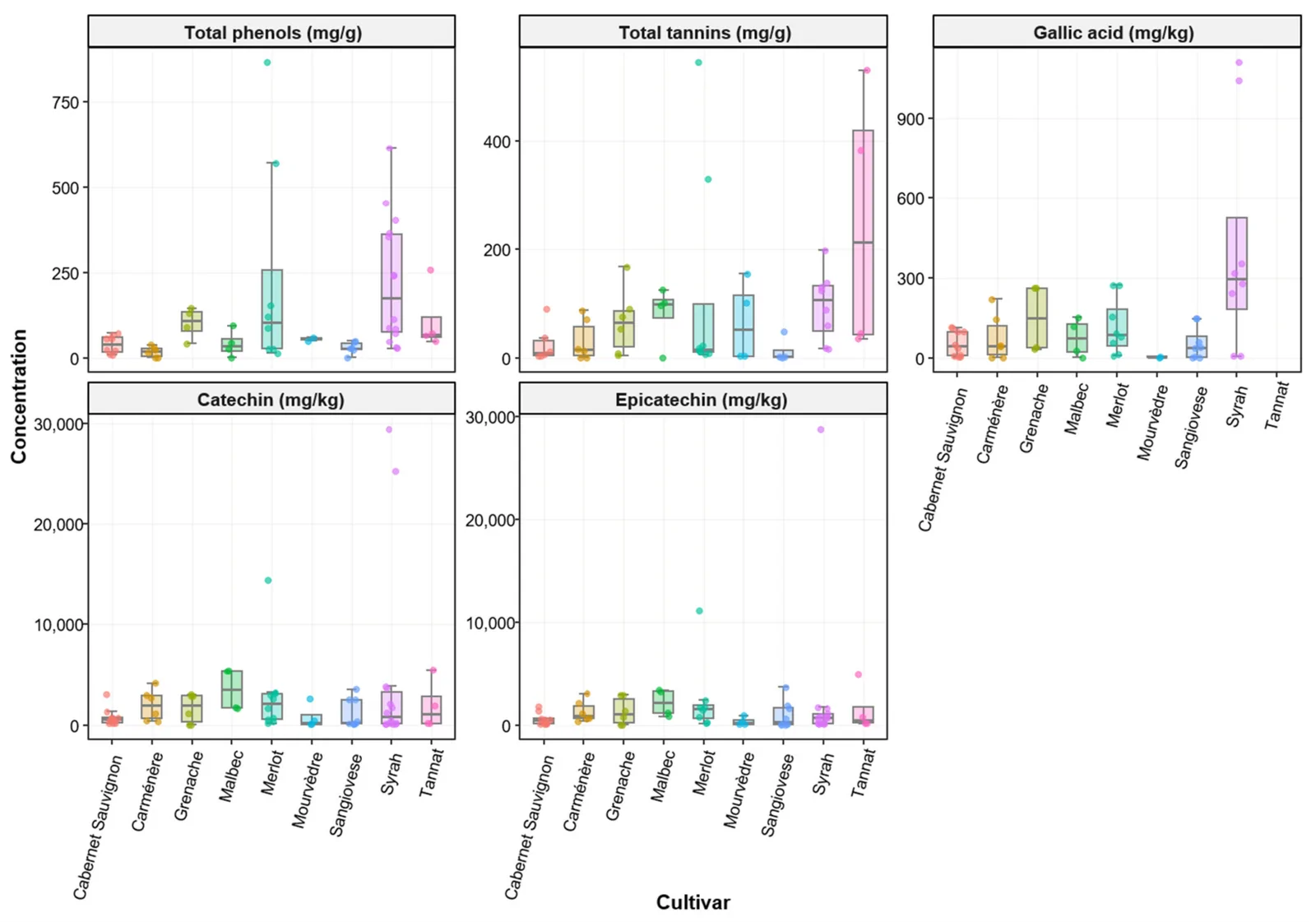
Box plots showing phenolic compound concentrations in the seeds of nine *Vitis vinifera* L. cultivars: total phenols, total tannins, and gallic acid (**upper** panels), and catechin and epicatechin (**lower** panels). Each box represents the interquartile range (IQR), the horizontal line indicates the median, and whiskers extend to 1.5 × IQR (Tukey’s criterion). Points represent individual values from each compiled study. Color denotes cultivar only and carries no additional statistical information.

**Figure 2:**
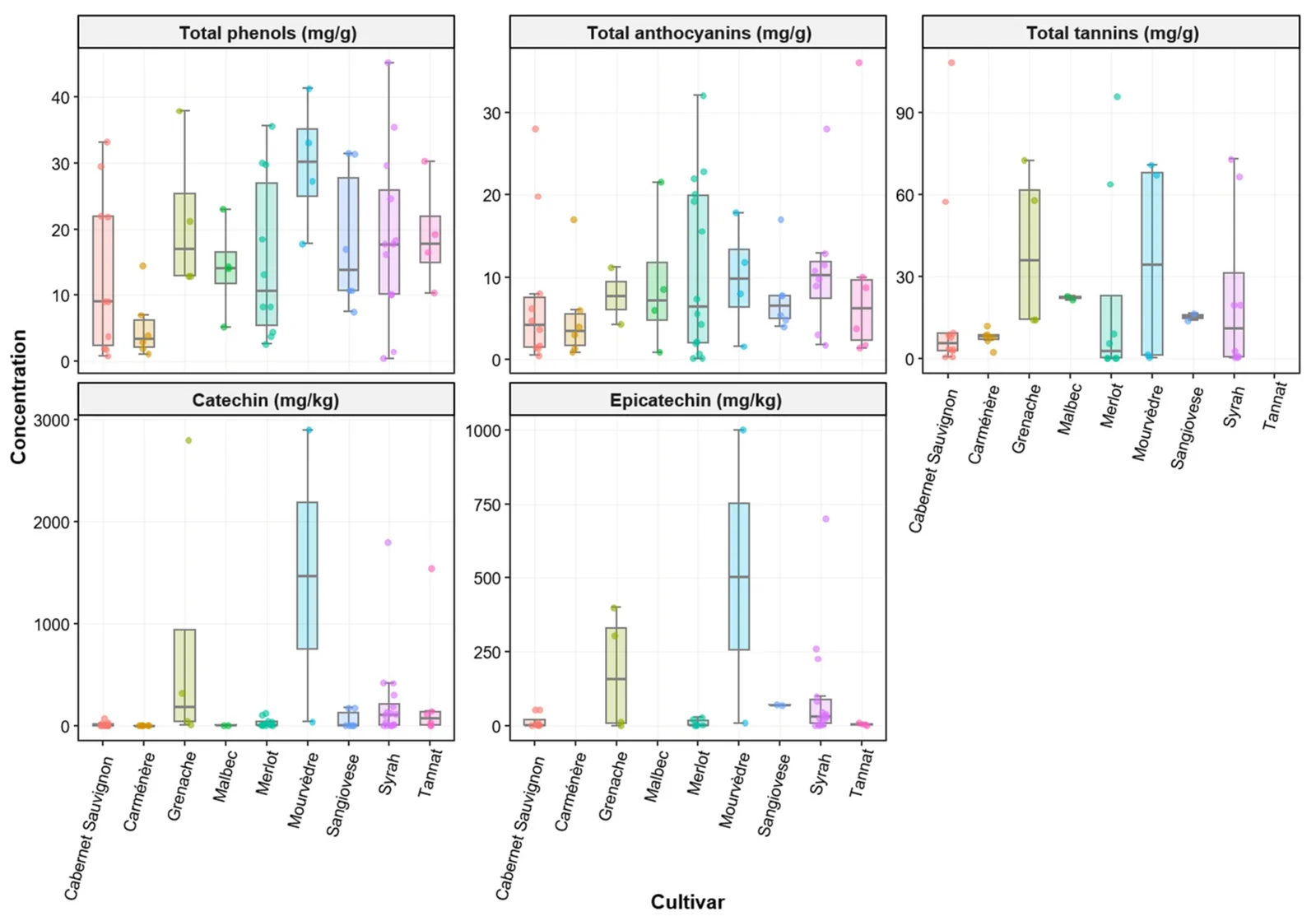
Box plots showing the phenolic compound concentrations in the skins of nine *Vitis vinifera* L. cultivars: total phenols, total anthocyanins, and total tannins (**upper** panels), and catechin and epicatechin (**lower** panels). Each box represents the interquartile range (IQR), the horizontal line indicates the median, and whiskers extend to 1.5 × IQR (Tukey’s criterion). Points represent individual values from each compiled study. Color denotes cultivar only and carries no additional statistical information.

**Figure 3:**
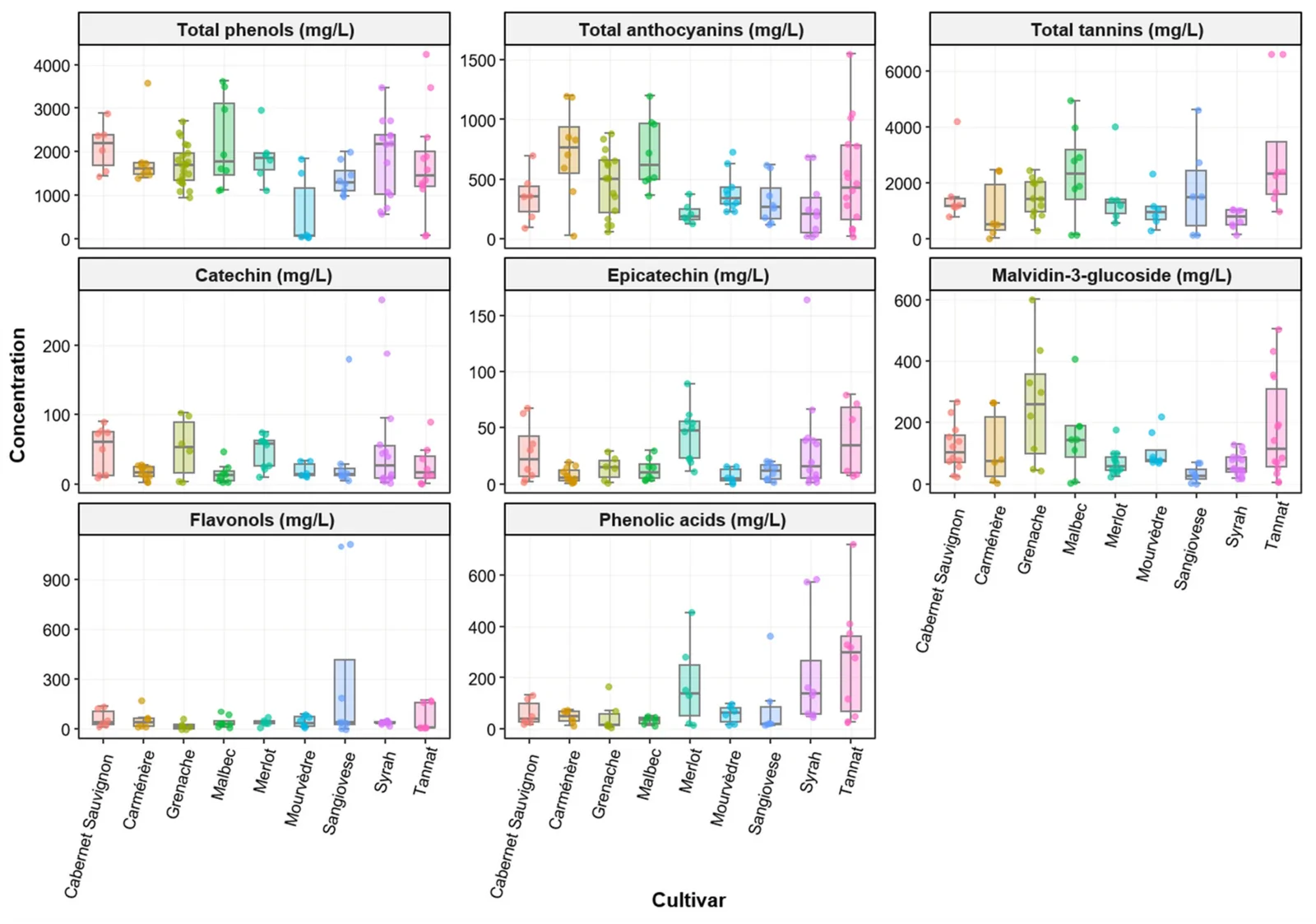
Box plots showing the phenolic compound concentrations in wines from nine *Vitis vinifera* L. cultivars: total phenols, anthocyanins, and tannins (**upper** panels); catechin, epicatechin, and malvidin-3-glucoside (**middle** panels); and flavonols and phenolic acids (**lower** panels). Each box represents the interquartile range (IQR), the horizontal line indicates the median, and whiskers extend to 1.5 × IQR (Tukey’s criterion). Points represent individual values from each compiled study. Color denotes cultivar only and carries no additional statistical information.

## Data Availability

The original contributions presented in this study are included in the article/Supplementary Material. Further inquiries can be directed to the corresponding author.

## Supplementary Materials

The following supporting information can be downloaded at: https://www.mdpi.com/article/10.3390/antiox15101355/s1, Table S1. Concentration range of phenolic compounds in seeds of varieties of *Vitis vinifera* L. [7,9,14,38,71,91,102,103,104,105,106,107,108,109,110,111,112,113,114,115,116,117,118,119,120,121,122,123,124]; Table S2. Concentration range of phenolic compounds in skins of varieties of *Vitis vinifera* L. [3,7,14,24,102,103,105,107,119,120,123,124,125,126,127,128,129,130,131,132,133,134,135,136,137]; Table S3. Concentration range of phenolic compounds in wines of varieties of *Vitis vinifera* L. [6,104,118,119,120,125,130,136,138,139,140,141,142,143,144,145,146,147,148,149,150,151,152,153,154,155,156,157,158,159,160,161,162,163,164,165,166,167,168,169,170,171,172,173,174,175,176,177,178,179,180,181,182,183,184,185,186,187,188,189,190,191,192,193,194,195,196]; Table S4. Mean concentration, dispersion (SD) and coefficient of variation (CV) of phenolic compounds in seeds, skins and wine.
