# Supplementary material for: The High Dispersion of Phenolic Concentration in the Seeds, Skins and Wines of Vitis vinifera L. Cultivars Limits Comparative Analysis and Varietal Categorization Under Heterogeneous Conditions
Source: Antioxidants (Basel). 2026 Oct 20;15(10):1355. doi: 10.3390/antiox15101355 (PMC13701742; doi:10.3390/antiox15101355)
Supplement: Supplementary file 1 [file antioxidants-15-01355-s001.zip › antioxidants-4481087-supplementary.pdf]

Table S1. Concentration range of phenolic compounds in **seeds** of varieties of *Vitis vinifera* L.

	Total phenols (mg/g)				Total tannins (mg/g)			
**Cultivar**	**Min**	**Max**	**Reference**		**Min**	**Max**	**Reference**	
**Cabernet Sauvignon**	66.9	72.2	Pantelic et al. (2016a)	[102]	7.2	12.0	Obreque-Slier et al. (2012)	[9]
	10.2	23.8	Obreque-Slier et al. (2012)	[9]	3.4	3.8	Torchio et al. (2013)	[117]
	8.5	20.4	Obreque-Slier et al. (2010)	[7]	36.9	90.3	Obreque-Slier et al. (2010)	[7]
	55.9	57.3	Tahmaz Karaman et al. (2021)	[103]				
**Carménère**	15.1	28.5	Obreque-Slier et al. (2012)	[9]	13.6	16.4	Obreque-Slier et al. (2012)	[9]
	0.4	37.7	Obreque-Slier et al. (2023)	[104]	0.2	71.5	Obreque-Slier et al. (2023)	[104]
	0.4	22.5	Obreque-Slier et al. (2010)	[7]	0.2	86.9	Obreque-Slier et al. (2010)	[7]
**Grenache**	41.2	88.7	Ky et al. (2014)	[105]	89.9	167.8	Ky et al. (2014)	[105]
	129.9	145.3	Luzio et al. (2021)	[106]	4.08	8.22	Edo-Roca et al. (2014)	[118]
					53.7	75.6	Luzio et al. (2021)	[106]
**Malbec**	25.7	43.2	Fanzone et al. (2011)	[107]	96.8	125.2	Fanzone et al. (2011)	[107]
	0.4	93.7	Obreque-Slier et al. (2023)	[104]	0.2	101.4	Obreque-Slier et al. (2023)	[104]
**Merlot**	13.5	26.2	Obreque-Slier et al. (2012)	[9]	8.4	11.7	Obreque-Slier et al. (2012)	[9]
	571.0	867.0	Benbouguerra et al. (2020)	[108]	329.0	545.0	Benbouguerra et al. (2020)	[108]
	121.4	152.3	Ivanova et al. (2011)	[109]	18.2	23.1	Ivanova et al. (2011)	[109]
	26.6	88.0	Zhou et al. (2018)	[110]	6.4	13.0	Zhou et al. (2018)	[110]
**Mourvèdre**	49.7	59.6	Ky et al. (2014)	[105]	3.0	3.0	Torchio et al. (2013)	[117]
					101.0	154.9	Ky et al. (2014)	[105]
**Sangiovese**	45.8	48.9	Pantelic et al. (2016a)	[102]	1.8	2.1	Torchio et al. (2013)	[117]
	0.4	24.2	Obreque-Slier et al. (2023)	[104]	0.2	48.0	Obreque-Slier et al. (2023)	[104]
	27.0	27.7	Tahmaz Karaman et al. (2021)	[103]				
**Syrah**	112.0	365.0	Capozzi et al. (2024)	[111]	89.1	123.3	Ky et al. (2014)	[105]
	47.4	72.8	Ky et al. (2014)	[105]	131.0	138.6	Georgieva et al. (2026)	[112]
	241.0	243.8	Georgieva et al. (2026)	[112]	18.0	198.0	Benbouguerra et al. (2020)	[108]
	27.7	31.5	Favre et al. (2024)	[113]	16.8	59.2	Barreto de Oliveira (2019b)	[119]
	355.0	405.0	Tita et al. (2021)	[114]				
	454.0	615.0	Benbouguerra et al. (2020)	[108]				
	84.8	86.4	Dabetic et al. (2022)	{115]				
**Tannat**	72	258	Benbouguerra et al. (2020)	[108]	382	530	Benbouguerra et al. (2020)	[108]
	47.87	65.29	Dudoit et al. (2020)	[116]	35.24	45.45	Dudoit et al. (2020)	[116]

	Gallic acid (mg/kg)				Catechin (mg/kg)			
**Cultivar**	**Min**	**Max**	**Reference**		**Min**	**Max**	**Reference**	
**Cabernet**	98.3	98.3	Pantelic et al. (2016a)	[102]	691.1	691.1	Pantelic et al. (2016a)	[102]

**Sauvignon**	50.0	110.0	Obreque-Slier et al. (2012)	[9]	440.0	1333.3	Obreque-Slier et al. (2012)	[9]
	1.7	4.7	Torchio et al. (2013)	[117]	135.1	143.4	Torchio et al. (2013)	[117]
	36.9	113.2	Obreque-Slier et al. (2010)	[7]	702.0	3045.0	Obreque-Slier et al. (2010)	[7]
	7.5	10.5	Rodríguez Montealegre et al. (2006)	[14]	190.0	360.0	Rodríguez Montealegre et al. (2006)	[14]
**Carménère**	46.7	143.3	Obreque-Slier et al. (2012)	[9]	1153.3	2700.0	Obreque-Slier et al. (2012)	[9]
	0.7	41.2	Obreque-Slier et al. (2023)	[104]	379.2	4160.8	Obreque-Slier et al. (2023)	[104]
	0.7	220.0	Obreque-Slier et al. (2010)	[7]	467.3	2952.0	Obreque-Slier et al. (2010)	[7]
**Grenache**	261.8	261.8	Guchu et al. (2015)	[120]	35.5	42.1	Torchio et al. (2013)	[117]
	32.5	37.9	Guendez et al. (2005)	[121]	1100.0	3000.0	Ky et al. (2014)	[105]
					2866.6	2866.6	Guchu et al. (2015)	[120]
**Malbec**	26.9	151.6	Fanzone et al. (2011)	[107]	1662.9	5345.2	Fanzone et al. (2011)	[107]
	0.7	118.1	Obreque-Slier et al. (2023)	[104]	1758.4	5333.1	Obreque-Slier et al. (2023)	[104]
**Merlot**	80.0	153.3	Obreque-Slier et al. (2012)	[9]	1645.0	2923.3	Obreque-Slier et al. (2012)	[9]
	56.2	92.2	Banjanin et al. (2019)	[122]	2602.0	14362.0	Chira et al. (2009)	[38]
	271.0	273.0	Guendez et al. (2005)	[121]	513.0	713.0	Banjanin et al. (2019)	[122]
	7.68	11.92	Rodríguez Montealegre et al. (2006)	[14]	3111.0	3209.0	Tahmaz Karaman et al. (2021)	[103]
					142	338	Rodríguez Montealegre et al. (2006)	[14]
**Mourvèdre**	1.2	4.4	Torchio et al. (2013)	[117]	60.4	67.8	Torchio et al. (2013)	[117]
					430.0	2600.0	Ky et al. (2014)	[105]
**Sangiovese**	0.7	58.9	Obreque-Slier et al. (2023)	[104]	133.8	133.8	Pantelic et al. (2016a)	[102]
	147.4	147.4	Pantelic et al. (2016a)	[102]	53.6	62.4	Torchio et al. (2013)	[117]
	0.2	2.7	Torchio et al. (2013)	[117]	386.1	3536.8	Obreque-Slier et al. (2023)	[104]
	35.4	38.6	Guendez et al. (2005)	[121]	2502.0	2544.0	Tahmaz Karaman et al. (2021)	[103]
**Syrah**	1040.0	1110.0	Georgieva et al. (2026)	[112]	46.32	207.21	Barreto de Oliveira (2019b)	[119]
	278.0	353.9	Macías-Gallardo et al. (2025)	[123]	25200	29400	Capozzi et al. (2024)	[111]
	5.5	8.2	Rodríguez Montealegre et al. (2006)	[14]	1705	2086	Georgieva et al. (2026)	[112]
	241.0	318.0	Dabetic et al. (2022)	[115]	87.776	101.394	Macías-Gallardo et al. (2025)	[123]
					3665.0	3845.0	Tahmaz Karaman et al. (2021)	[103]
					101.0	139.0	Rodríguez Montealegre et al. (2006)	[14]
					469.0	1250.0	Dabetic et al. (2022)	[115]
**Tannat**					1942.4	5462.4	Dudoit et al. (2020)	[116]
					146.0	160.0	Boido et al. (2011)	[125]

**Epicatechin (mg/kg)**				
**Cultivar**	**Min**	**Max**	**Reference**	
**Cabernet**	511.5	511.5	Pantelic et al. (2016a)	[102]
**Sauvignon**	626.7	1343.3	Obreque-Slier et al. (2012)	[9]
	78.2	85.5	Torchio et al. (2013)	[117]
	360.4	1756.0	Obreque-Slier et al. (2010)	[7]
	105.0	155.0	Rodríguez Montealegre et al. (2006)	[14]

**Carménère**	576.7	1136.7	Obreque-Slier et al. (2012)	[9]
	676.2	3045.0	Obreque-Slier et al. (2023)	[104]
	378.9	2115.0	Obreque-Slier et al. (2010)	[7]
**Grenache**	23.3	24.0	Torchio et al. (2013)	[117]
	2903.0	2903.0	Guchu et al. (2015)	[120]
	790.0	1400.0	Ky et al. (2014)	[105]
**Malbec**	835.9	3187.4	Fanzone et al. (2011)	[107]
	1198.4	3397.3	Obreque-Slier et al. (2023)	[104]
**Merlot**	755.0	1416.7	Obreque-Slier et al. (2012)	[9]
	2411.0	11156.0	Chira et al. (2009)	[38]
	1669.0	1739.0	Tahmaz Karaman et al. (2021)	[103]
	138.0	282.0	Rodríguez Montealegre et al. (2006)	[14]
**Mourvèdre**	47.2	58.6	Torchio et al. (2013)	[117]
	380.0	900.0	Ky et al. (2014)	[105]
**Sangiovese**	19.4	19.4	Pantelic et al. (2016a)	[102]
	60.9	92.2	Torchio et al. (2013)	[117]
	589.7	3664.2	Obreque-Slier et al. (2023)	[104]
	1587.0	1857.0	Tahmaz Karaman et al. (2021)	[103]
**Syrah**	129.9	775.3	Barreto de Oliveira (2019b)	[119]
	700.0	28800.0	Capozzi et al. (2024)	[111]
	671.0	848.0	Georgieva et al. (2026)	[112]
	132.1	154.6	Macías-Gallardo et al. (2025)	[123]
	1634.0	1740.0	Tahmaz Karaman et al. (2021)	[103]
	96.0	164.0	Rodríguez Montealegre et al. (2006)	[14]
	121.0	1080.0	Dabetic et al. (2022)	[115]
**Tannat**	730.2	4944.3	Dudoit et al. (2020)	[116]
	169.0	213.0	Boido et al. (2011)	[125]

Table S2. Concentration range of phenolic compounds in **skins** of varieties of *Vitis vinifera* L.

	Total phenols (mg/g)				Total anthocyanins (mg/g)			
**Cultivar**	**Min**	**Max**	**Reference**		**Min**	**Max**	**Reference**	
**Cabernet Sauvignon**	0.8	1.8	Obreque-Slier et al. (2010)	[7]	0.5	1.3	Obreque-Slier et al. (2010)	[7]
	1.9	3.8	Obreque-Slier et al. (2013)	[3]	1.5	3.7	Obreque-Slier et al. (2013)	[3]
	9.1	9.1	Pantelić et al. (2016a)	[102]	8.0	28.0	Wei et al. (2023)	[132]
	29.5	33.2	Lorrain et al. (2011)	[24]	4.7	19.8	Lorrain et al. (2011)	[24]
	21.9	22.0	Tahmaz Karaman et al. (2021)	[103]	1.7	6.2	Ortega-Regules et al. (2008)	[133]
**Carménère**	1.1	2.9	Obreque-Slier et al. (2010)	[7]	6.0	17.0	Obreque-Slier et al. (2021)	[126]
	7.0	14.5	Obreque-Slier et al. (2021)	[126]	0.9	3.0	Obreque-Slier et al. (2010)	[7]
	1.9	3.9	Obreque-Slier et al. (2013)	[3]	1.3	4.0	Obreque-Slier et al. (2013)	[3]
**Grenache**	21.2	37.9	Ky et al. (2014)	[105]	4.3	11.2	Ky et al. (2014)	[105]
	12.9	12.9	Somkuwar et al. (2019)	[127]				
**Malbec**	14.0	23.0	Obreque-Slier et al. (2021)	[126]	8.5	21.5	Obreque-Slier et al. (2021)	[126]
	5.2	14.3	Fanzone et al. (2011)	[107]	0.9	6.0	Fanzone et al. (2011)	[107]
**Merlot**	2.6	4.4	Obreque-Slier et al. (2013)	[3]	2.2	4.3	Obreque-Slier et al. (2013)	[3]
	8.3	8.3	Pantelić et al. (2016a)	[102]	7.4	22.8	Lorrain et al. (2011)	[24]
	30.0	35.6	Lorrain et al. (2011)	[24]	15.6	20.1	Anić et al. (2021)	[134]
	13.1	18.5	Benbouguerra et al. (2021)	[128]	19.2	32.1	Shi et al. (2018)	[135]
	3.7	29.8	Pantelić et al. (2016b)	[129]	2.0	22.0	Benbouguerra et al. (2020)	[108]
					0.2	5.6	Ortega-Regules et al. (2008)	[133]
					0.1	0.7	Pantelić et al. (2016b)	[129]
**Mourvèdre**	17.8	33.1	Moreno-Olivares et al. (2022)	[130]	1.6	8.0	Ortega-Regules et al. (2008)	[133]
	27.3	41.3	Ky et al. (2014)	[105]	11.8	17.8	Ky et al. (2014)	[105]
**Sangiovese**	7.5	17.0	Obreque-Slier et al. (2021)	[126]	4.0	17.0	Obreque-Slier et al. (2021)	[126]
	10.7	10.7	Pantelić et al. (2016a)	[102]	4.8	5.4	Filippetti et al. (2013)	[136]
	31.3	31.5	Tahmaz Karaman et al. (2021)	[103]	7.7	7.8	Tahmaz Karaman et al. (2021)	[103]
**Syrah**	10.1	10.1	Pantelić et al. (2016a)	[102]	3.0	28.0	Benbouguerra et al. (2020)	[108]
	16.2	18.3	Benbouguerra et al. (2021)	[128]	10.8	12.9	Ky et al. (2014)	[105]
	0.4	1.4	Barreto de Oliveira (2019b)	[119]	1.8	9.8	Ortega-Regules et al. (2008)	[133]
	35.5	45.2	Ky et al. (2014)	[105]	8.9	11.5	da Mota et al. 2011	[131]
	17.7	17.7	Somkuwar et al. (2019)	[127]				
	24.7	29.7	da Mota et al. 2011	[131]				
**Tannat**	16.5	19.2	Benbouguerra et al. (2021)	[128]	10.0	36.0	Benbouguerra et al. (2020)	[108]
	10.3	30.3	Dudoit et al. (2020)	[116]	1.4	8.7	Dudoit et al. (2020)	[116]
					1.8	3.8	Boido et al. (2011)	[125]

	Total tannins (mg/g)				Catechin (mg/kg)			
**Cultivar**	**Min**	**Max**	**Reference**		**Min**	**Max**	**Reference**	
**Cabernet Sauvignon**	2.9	7.9	Obreque-Slier et al. (2010)	[7]	0.5	5.1	Obreque-Slier et al. (2010)	[7]
	3.5	9.3	Obreque-Slier et al. (2013)	[3]	1.2	6.1	Obreque-Slier et al. (2013)	[3]
	3.3	8.8	Wei et al. (2023)	[132]	31.4	71.2	Chen et al. (2020)	[137]
	57.4	108.2	Lorrain et al. (2011)	[24]	0.2	0.5	Wei et al. (2023)	[132]
	0.6	0.6	Ortega-Regules et al. (2008)	[133]	5.9	5.9	Pantelić et al. (2016a)	[102]
					9.3	24.7	Rodríguez Montealegre et al. (2006)	[14]
**Carménère**	2.5	8.7	Obreque-Slier et al. (2010)	[7]	1.3	3.1	Obreque-Slier et al. (2010)	[7]
	8.5	12.0	Obreque-Slier et al. (2021)	[126]	1.1	4.0	Obreque-Slier et al. (2013)	[3]
	6.6	7.9	Obreque-Slier et al. (2013)	[3]	0.8	2.1	Obreque-Slier et al. (2021)	[126]
**Grenache**	57.9	72.5	Ky et al. (2014)	[105]	10.0	2800.0	Ky et al. (2014)	[105]
	14.2	14.2	Somkuwar et al. (2019)	[127]	48.1	319.7	Guchu et al. (2015)	[120]
**Malbec**	21.5	23.0	Obreque-Slier et al. (2021)	[126]	5.0	5.7	Obreque-Slier et al. (2021)	[126]
**Merlot**	5.6	9.2	Obreque-Slier et al. (2013)	[3]	0.4	4.3	Obreque-Slier et al. (2013)	[3]
	63.8	95.9	Lorrain et al. (2011)	[24]	7.5	7.5	Pantelić et al. (2016a)	[102]
	0.1	0.2	Shi et al. (2018)	[135]	5.4	38.2	Shi et al. (2018)	[135]
	0.3	0.3	Ortega-Regules et al. (2008)	[133]	24.0	107.0	Benbouguerra et al. (2021)	[128]
					11.1	123.6	Pantelić et al. (2016b)	[129]
					7.1	42.9	Rodríguez Montealegre et al. (2006)	[14]
**Mourvèdre**	0.4	1.4	Ortega-Regules et al. (2008)	[133]	40.0	2900.0	Ky et al. (2014)	[105]
	67.2	70.8	Ky et al. (2014)	[105]				
**Sangiovese**	14.0	16.5	Obreque-Slier et al. (2021)	[126]	3.9	3.9	Pantelić et al. (2016a)	[102]
					0.0	0.0	Obreque-Slier et al. (2021)	[126]
					172.6	175.2	Tahmaz Karaman et al. (2021)	[103]
**Syrah**	0.86	2.81	Barreto de Oliveira (2019b)	[119]	5.4	5.4	Pantelić et al. (2016a)	[102]
	66.4	73	Ky et al. (2014)	[105]	39.8	111.1	Benbouguerra et al. (2021)	[128]
	0.4	0.4	Ortega-Regules et al. (2008)	[133]	2.6	8.2	Barreto de Oliveira (2019b)	[119]
	19.5	19.5	Somkuwar et al. (2019)	[127]	300.0	1800.0	Ky et al. (2014)	[105]
					104.0	187.0	Bustamante et al. (2026)	[15]
					121.0	134.3	Tahmaz Karaman et al. (2021)	[103]
					4.6	12.4	Rodríguez Montealegre et al. (2006)	[14]
					418.3	419.8	Macías-Gallardo et al. (2025)	[123]
**Tannat**					29.4	114.5	Benbouguerra et al. (2021)	[128]
					143.7	1539.4	Dudoit et al. (2020)	[116]
					4.4	5.5	Boido et al. (2011)	[125]

Epicatechin (mg/kg)				
**Cultivar**	**Min**	**Max**	**Reference**	
**Cabernet Sauvignon**	0.37	3.53	Chen et al. (2020)	[137]
	3.01	3.01	Pantelić et al. (2016a)	[102]
	54.07	54.09	Tahmaz Karaman et al. (2021)	[103]
	2.49	9.91	Rodríguez Montealegre et al. (2006)	[14]
**Grenache**	1.00	400.00	Ky et al. (2014)	[105]
	12.20	305.50	Guchu et al. (2015)	[120]
**Malbec**				
**Merlot**	1.38	5.90	Benbouguerra et al. (2021)	[128]
	0.67	28.15	Pantelić et al. (2016b)	[129]
	2.8	23.2	Rodríguez Montealegre et al. (2006)	[14]
**Mourvèdre**	10.00	1000.00	Ky et al. (2014)	[105]
**Sangiovese**	68.79	71.35	Tahmaz Karaman et al. (2021)	[103]
**Syrah**	3.02	3.02	Pantelić et al. (2016a)	[102]
	0.86	30.94	Benbouguerra et al. (2021)	[128]
	34.17	82.64	Barreto de Oliveira (2019b)	[119]
	100	700	Ky et al. (2014)	[105]
	227	261	Bustamante et al. (2026)	[15]
	24.74	24.76	Tahmaz Karaman et al. (2021)	[103]
	2.1	11.7	Rodríguez Montealegre et al. (2006)	[14]
	40.712	45.416	Macías-Gallardo et al. (2025)	[123]
**Tannat**	2.32	9.85	Benbouguerra et al. (2021)	[128]
	4.80	6.00	Boido et al. (2011)	[125]

Table S3. Concentration range of phenolic compounds in **wines** of varieties of *Vitis vinifera* L.

	Total phenols (mg/L)				Total anthocyanins (mg/L)			
**Cultivar**	**Min**	**Max**	**Reference**		**Min**	**Max**	**Reference**	
**Cabernet Sauvignon**	2029.0	2880.0	Van Leeuw et al. (2014)	[138]	466.6	696.4	Cáceres et al. (2018)	[6]
	2380.1	2398.3	Ju et al. (2021)	[139]	353.1	358.5	Ju et al. (2021)	[139]
	1439.0	1557.0	Cáceres et al. (2018)	[6]	94.4	189.2	Ren et al. (2023)	[166]
**Carménère**	1400.0	1690.0	Vazallo-Valleumbrocio et al. (2017)	[140]	595.0	705.0	Vazallo-Valleumbrocio et al. (2017)	[140]
	1569.0	3591.0	Zuñiga et al. (2014)	[141]	27.0	397.0	Zuñiga et al. (2014)	[141]
	1740.0	1760.0	López-Solis et al. (2024a)	[142]	1190.0	1200.0	López-Solis et al. (2024a)	[142]
	1480.0	1485.0	Medel-Marabolí et al. (2021)	[143]	830.0	850.0	Medel-Marabolí et al. (2021)	[143]
**Grenache**	1281.2	1293.6	López-Giral et al. (2015)	[144]	58.0	168.0	López-Giral et al. (2015)	[144]
	1705.8	1720.0	López-Giral et al. (2015)	[144]	110.0	112.0	Gombau et al. (2020)	[145]
	942.5	1815.4	Gombau et al. (2020)	[145]	657.8	840.3	Maza et al. (2019)	[146]
	1497.3	2370.2	Maza et al. (2019)	[146]	236.0	506.0	Pascual et al. (2016)	[147]
	1087.4	1359.5	Edo-Roca et al. (2014)	[118]	356.0	619.0	López-Giral et al. (2022)	[149]
	1451.4	2444.4	Pascual et al. (2016)	[147]	630.0	668.0	Luengo et al. (2014)	[150]
	2161.7	2698.9	Diago et al. (2010)	[148]	382.0	505.3	López et al. (2008)	[151]
	1098.0	1720.0	López-Giral et al. (2022)	[149]	749.0	883.7	Maza et al. (2020)	[152]
	1917.9	1981.5	Luengo et al. (2014)	[150]				
	1341.8	1571.5	López et al. (2008)	[151]				
	1776.5	2177.6	Maza et al. (2020)	[152]				
**Malbec**	1932.0	3506.8	Fanzone et al. (2010)	[153]	364.2	519.4	Fanzone et al. (2013)	[154]
	2988.9	3624.2	Fanzone et al. (2013)	[154]	490.0	500.8	Medel-Marabolí et al. (2021)	[143]
	1120.0	1137.7	Medel-Marabolí et al. (2021)	[143]	960.0	980.0	López-Solis et al. (2024a)	[142]
	1580.0	1620.0	López-Solis et al. (2024a)	[142]	720.0	1200.0	Casassa et al. (2022)	[167]
**Merlot**	1940.0	2962.0	Van Leeuw et al. (2014)	[138]	205.9	373.6	Osrecak et al. (2016)	[155]
	1510.0	1808.0	Osrecak et al. (2016)	[155]	178.0	265.0	Ntuli et al. (2023)	[156]
	1114.0	1970.0	Ntuli et al. (2023)	[156]	125.3	158.5	Li et al. (2025)	[168]
**Mourvèdre**	1522.0	1836.0	Moreno-Olivares et al. (2022)	[130]	437.0	633.0	Moreno-Olivares et al. (2022)	[130]
	24.0	56.3	Gómez-Plaza et al. (2016)	[157]	229.2	304.3	Álvarez et al. (2006)	[169]
	56.7	92.8	Gómez-Plaza et al. (2016)	[157]	284.0	729.0	Martínez et al. (2021)	[170]
					304.0	380.0	Romero et al. (2013)	[171]
					231.0	404.2	Romero et al. (2015)	[172]
**Sangiovese**	1123.0	1826.0	Filippetti (2013)	[136]	260.0	280.6	Medel-Marabolí et al. (2021)	[143]
	980.0	1016.7	Medel-Marabolí et al. (2021)	[143]	600.0	620.0	López-Solis et al. (2024a)	[142]
	1280.0	1330.0	López-Solis et al. (2024a)	[142]	118.8	362.9	Rinaldi et al. (2020)	[173]
	1470.0	2005.0	Becatti et al. (2014)	[158]	138.0	178.0	Becatti et al. (2014)	[158]
**Syrah**	1068.5	2313.4	Oliveira et al. (2019b)	[119]	190.8	375.6	Oliveira et al. (2019b)	[119]

	1763.0	3476.0	Van Leeuw et al. (2014)	[138]	684.8	688.1	Ju et al. (2021)	[139]
	2374.6	2387.1	Ju et al. (2021)	[139]	20.2	21.5	Nascimiento-Alves et al. (2022)	[160]
	2711.1	2711.1	Gordillo et al. (2021b)	[159]	37.0	86.0	Bian et al. (2026)	[161]
	563.0	630.7	Nascimiento-Alves et al. (2022)	[160]	232.3	244.7	da Mota et al. 2011	[131]
	720.0	1020.0	Bian et al. (2026)	[161]				
	2170.0	2190.0	da Mota et al. 2011	[131]				
**Tannat**	1227.0	1586.0	Favre et al. (2014)	[162]	281.0	347.0	Favre et al. (2014)	[162]
	1893.0	4246.0	Vidal et al. (2017)	[163]	18.2	461.8	Vidal et al. (2018)	[174]
	1361.0	1859.0	González-Neves et al. (2015)	[164]	408.0	781.0	González-Neves et al. (2015)	[164]
	2345.5	3472.8	Piccardo et al. (2019)	[165]	794.0	1054.0	Piccardo et al. (2019)	[165]
	1154.9	1270.9	Nascimiento-Alves et al. (2022)	[160]	82.6	85.5	Nascimiento-Alves et al. (2022)	[160]
	74.0	98.0	Boido et al. (2011)	[125]	1016.0	1550.0	Boido et al. (2011)	[125]
					64.3	184.0	de Andrade et al. (2013)	[175]
					516.0	553.2	González-Neves et al. (2007)	[176]

	Total tannins (mg/L)				Catechin (mg/L)			
**Cultivar**	**Min**	**Max**	**Reference**		**Min**	**Max**	**Reference**	
**Cabernet Sauvignon**	1177.1	1188.6	Ju et al. (2021)	[139]	50.72	74.93	Van Leeuw et al. (2014)	[138]
	781.2	1149.3	Cáceres et al. (2018)	[6]	8.93	89.69	Belmiro et al. (2017)	[179]
	1500.0	4200.0	Song et al. (2024)	[177]	12.21	12.67	Tong et al. (2023)	[180]
					72.9	76.9	Lingua et al. (2016)	[181]
**Carménère**	14.0	241.0	Zuñiga et al. (2014)	[141]	2.3	2.4	Vazallo-Valleumbrocio et al. (2017)	[140]
	2420.0	2450.0	López-Solis et al. (2024a)	[142]	8.7	24.5	Zuñiga et al. (2014)	[141]
	520.0	530.0	Medel-Marabolí et al. (2021)	[143]	22.5	25.2	Valentin et al. (2020)	[182]
					21	27.8	López-Solis et al. (2024a)	[142]
					12.6	14.0	Medel-Marabolí et al. (2021)	[143]
					11.3	27.2	López-Solis et al. (2024b)	[183]
**Grenache**	840.0	1979.0	Gombau et al. (2020)	[145]	98.9	102.8	Luengo et al. (2014)	[150]
	987.6	1458.4	Maza et al. (2019)	[146]	48.19	58.5	Guchu et al. (2015)	[120]
	300.0	839.0	Pascual et al. (2016)	[147]	2.9	4.4	López-Giral et al. (2015)	[144]
	1433.0	2460.0	López-Giral et al. (2022)	[149]				
	2100.0	2200.0	Luengo et al. (2014)	[150]				
	1207.1	2015.9	Maza et al. (2020)	[152]				
**Malbec**	2782.9	4943.3	Fanzone et al. (2010)	[153]	24.4	47	Fanzone et al. (2010)	[153]
	2905.5	3990.1	Fanzone et al. (2013)	[154]	12	13.8	Medel-Marabolí et al. (2021)	[143]
	150.0	145.2	Medel-Marabolí et al. (2021)	[143]	2.1	2.2	da Costa et al. (2021)	[184]
	1800.0	1890.0	López-Solis et al. (2024a)	[142]	5.1	16.9	Obreque-Slier et al. (2023)	[104]

**Merlot**	1400.0	4000.0	Song et al. (2024)	[177]	26.19	72.17	Van Leeuw et al. (2014)	[138]
	1191.1	1395.8	Li et al. (2025)	[168]	25.9	61.1	Osrecak et al. (2016)	[155]
	566.0	824.0	Casassa et al. (2021)	[178]	21	62	Ntuli et al. (2023)	[156]
					9.92	74.6	Marquez et al. (2012)	[185]
					56.9	61.9	Lingua et al. (2016)	[181]
**Mourvèdre**	628.0	838.0	Moreno-Olivares et al. (2022)	[130]	11.8	12.3	Bautista-Ortín et al. (2007)	[187]
	302.0	1159.8	Gómez-Plaza et al. (2016)	[157]	33.5	33.5	Bautista-Ortín et al. (2007)	[187]
	1072.4	2334.2	Gómez-Plaza et al. (2016)	[157]	9.6	15.8	Bautista-Ortín et al. (2007)	[187]
**Sangiovese**	130.0	139.7	Medel-Marabolí et al. (2021)	[143]	11.4	12.8	Medel-Marabolí et al. (2021)	[143]
	1500.0	1510.0	López-Solis et al. (2024a)	[142]	180.8	19.3	Tessarin et al. (2016)	[187]
	2740.0	4610.0	Rinaldi et al. (2020)	[173]	4.8	15.5	Parpinello et al. (2015)	[188]
					11.9	28.6	Becatti et al. (2014)	[158]
**Syrah**	443.5	1037.7	Oliveira et al. (2019b)	[119]	3.3	8.6	Oliveira et al. (2019b)	[119]
	1027.8	1041.7	Ju et al. (2021)	[139]	46.55	56.88	Van Leeuw et al. (2014)	[138]
	620.0	1000.0	Casassa et al. (2022)	[167]	14.07	94.63	Belmiro et al.. 2017	[179]
	145.0	500.0	Bian et al. (2026)	[161]	39.8	44	Lingua et al.. 2016	[181]
					188.49	266.53	Gordillo et al. (2021b)	[159]
					9.23	10.35	Nascimiento-Alves et al. (2022)	[160]
					1.88	8.69	Bian et al. (2026)	[161]
**Tannat**	1653.0	2257.0	Favre et al. (2014)	[162]	0.57	1.55	Favre et al. (2014)	[162]
	2400.0	6600.0	Vidal et al. (2018)	[174]	22	89.3	Vidal et al. (2018)	[174]
	2400.0	6600.0	Vidal et al. (2017)	[163]	11.25	12.63	Nascimiento-Alves et al. (2022)	[160]
	977.0	1458.0	González-Neves et al. (2015)	[164]	37	49	Boido et al. (2011)	[125]

	**Epicatechin (mg/L)**				**Malvidin-3-glucoside (mg/L)**			
**Cultivar**	**Min**	**Max**	**Reference**		**Min**	**Max**	**Reference**	
**Cabernet Sauvignon**	13.1	35.8	Van Leeuw et al. (2014)	[138]	27.3	138.9	Van Leeuw et al. (2014)	[138]
	1.8	30.7	Belmiro et al. (2017)	[179]	153.2	175.9	Cáceres et al. (2018)	[6]
	4.3	7.5	Tong et al. (2023)	[180]	234.8	269.3	Cáceres et al. (2018)	[6]
	63.5	67.7	Lingua et al. (2016)	[181]	74.5	85.5	Tong et al. (2023)	[180]
					57.9	77.3	Lingua et al. (2016)	[181]
					23.1	124.0	de Andrade et al. (2013)	[175]
**Carménère**	1.7	1.9	Vazallo-Valleumbrocio et al. (2017)	[140]	79.5	70.4	Valentin et al. (2020)	[182]
	0.6	15.0	Zuñiga et al. (2014)	[141]	264.3	264.5	López-Solis et al. (2024a)	[142]
	16.4	19.3	Valentin et al. (2020)	[182]	3.7	10.7	Obreque-Slier et al. (2023)	[104]
	7.2	7.8	López-Solis et al. (2024a)	[142]				
	3.6	4.2	Medel-Marabolí et al. (2021)	[143]				
	5.1	11.3	López-Solis et al. (2024b)	[183]				
**Grenache**	14.4	15.9	Luengo et al. (2014)	[150]	300.4	331.6	Luengo et al. (2014)	[150]
	22.8	29.2	Guchu et al. (2015)	[120]	436.7	603.1	Maza et al. (2020)	[152]

	1.2	3.1	López-Giral et al. (2015)	[144]	43.7	47.6	Guchu et al. (2015)	[120]
**Malbec**	14.5	23.6	Fanzone et al. (2010)	[153]	189.9	408.8	Fanzone et al. (2010)	[153]
	4.3	4.9	Medel-Maraboli et al. (2021)	[143]	144.9	144.9	Medel-Maraboli et al. (2021)	[143]
	15.3	29.7	Urvieta et al. (2018)	[189]	112.5	188.3	Ren et al. (2023)	[166]
	2.8	6.8	Obreque-Slier et al. (2023)	[104]	3.8	9.3	Obreque-Slier et al. (2023)	[104]
**Merlot**	11.1	49.8	Van Leeuw et al. (2014)	[138]	50.1	99.4	Van Leeuw et al. (2014)	[138]
	22.9	23.0	Osrecak et al. (2016)	[155]	76.1	176.3	Osrecak et al. (2016)	[155]
	19.5	62.0	Ntuli et al. (2023)	[156]	70.0	82.0	Ntuli et al. (2023)	[156]
	46.7	89.5	Marquez et al. (2012)	[185]	23.5	99.7	Marquez et al. (2012)	[185]
	54.8	56.4	Lingua et al. (2016)	[181]	44.5	48.7	Lingua et al. (2016)	[181]
					36.6	40.5	de Andrade et al. (2013)	[175]
**Mourvèdre**	4.8	5.6	Bautista-Ortín et al. (2007)	[187]	167.4	219.3	Álvarez et al. (2006)	[169]
	15.4	15.6	Bautista-Ortín et al. (2007)	[187]	69.1	73.7	Ren et al. (2023)	[166]
	0.0	1.9	Bautista-Ortín et al. (2007)	[187]	72.7	89.1	Romero et al. (2015)	[172]
					76.8	79.9	Bautista-Ortín et al. (2007)	[187]
**Sangiovese**	4.2	4.6	Medel-Maraboli et al. (2021)	[143]	69.8	69.8	Medel-Maraboli et al. (2021)	[143]
	14.9	15.9	Tessarin et al. (2016)	[187]	22.6	40.9	Parpinello et al. (2015)	[188]
	1.6	9.5	Parpinello et al. (2015)	[188]	21.7	32.0	Becatti et al. (2014)	[158]
	19.5	20.2	Becatti et al. (2014)	[158]	1.6	3.8	Obreque-Slier et al. (2023)	[104]
**Syrah**	5.0	12.6	Oliveira et al. (2019b)	[119]	41.0	122.3	Van Leeuw et al. (2014)	[138]
	19.6	39.4	Van Leeuw et al. (2014)	[138]	47.2	128.7	Gómez-Míguez et al. (2007)	[190]
	4.5	35.9	Belmiro et al. (2017)	[179]	86.7	88.1	Lingua et al. (2016)	[181]
	39.2	41.6	Lingua et al. (2016)	[181]	22.7	79.7	de Andrade et al. (2013)	[175]
	66.4	164.8	Gordillo et al. (2021b)	[159]	45.5	52.0	Gordillo et al. (2021a)	[191]
	5.5	8.4	Nascimiento-Alves et al. (2022)	[160]	49.1	80.5	Gordillo et al. (2021b)	[159]
	1.4	1.5	Bian et al. (2026)	[161]	17.5	18.7	Nascimiento-Alves et al. (2022)	[160]
					42.4	79.6	Bian et al. (2026)	[161]
**Tannat**	11.6	79.7	Vidal et al. (2018)	[174]	143.0	194.6	Favre et al. (2014)	[162]
	7.2	8.9	Nascimiento-Alves et al. (2022)	[160]	4.5	188.4	Vidal et al. (2018)	[174]
	58.0	72.0	Boido et al. (2011)	[125]	71.3	73.7	Nascimiento-Alves et al. (2022)	[160]
					8.3	86.5	de Andrade et al. (2013)	[175]
					349.5	355.9	González-Neves et al. (2007)	[176]
					34.1	50.8	González-Neves et al. (2007)	[176]
					434.0	506.0	Boido et al. (2011)	[125]
	**Flavonols (mg/L)**				**Phenolic acids (mg/L)**			
**Cultivar**	**Min**	**Max**	**Reference**		**Min**	**Max**	**Reference**	
**Cabernet Sauvignon**	35.3	49.5	Van Leeuw et al. (2014)	[138]	118.0	133.0	Van Leeuw et al. (2014)	[138]
	122.3	137.2	Tong et al. (2023)	[180]	19.5	27.4	Ren et al. (2023)	[166]
	12.4	23.8	Ren et al. (2023)	[166]	31.8	50.7	Shi et al. (2026)	[194]
**Carménère**	15.5	20.7	Vazallo-Valleumbrocio et al. (2017)	[140]	69.99	74.39	Mendoza et al. (2011)	[195]

	62.9	67.9	Medel-Maraboli et al. (2021)	[143]	13.5	30.0	Zuñiga et al. (2014)	[141]
	14.4	171.3	Vergara et al. (2011)	[192]	38.2	67.7	Obreque-Slier et al. (2023)	[104]
**Grenache**	15.0	64.0	Pascual et al. (2016)	[147]	72.0	167.0	Pascual et al. (2016)	[147]
	20.7	26.4	Luengo et al. (2014)	[150]	12.5	14.5	Guchu et al. (2015)	[120]
	0.0	0.2	Leborgne et al. (2023)	[193]	7.3	21.2	López-Giral et al. (2015)	[144]
**Malbec**	11.2	16.7	Fanzone et al. (2010)	[153]	13.1	18.3	Ren et al. (2023)	[166]
	87.1	106.2	Fanzone et al. (2013)	[154]	35.0	40.0	Fanzone et al. (2010)	[153]
	32.2	35.0	Medel-Maraboli et al. (2021)	[143]	46.9	50.5	Fanzone et al. (2012)	[1]
	28.7	32.7	Ren et al. (2023)	[166]				
**Merlot**	33.9	41.9	Van Leeuw et al. (2014)	[138]	132.5	151.3	Van Leeuw et al. (2014)	[138]
	10.9	32.7	Ren et al. (2023)	[166]	15.3	24.3	Ren et al. (2023)	[166]
	49.5	70.4	Li et al. (2025)	[168]	282.4	454.8	Xie et al. (2023)	[196]
**Mourvèdre**	50.6	75.9	Romero et al. (2013)	[171]	17.7	19.2	Ren et al. (2023)	[166]
	72.7	89.1	Romero et al. (2015)	[172]	57.9	86.8	Pardo-García et al. (2014)	[124]
	19.3	22.1	Ren et al (2023)	[166]	69.7	97.9	Pardo-García et al. (2014)	[124]
	9.6	17.6	Bautista-Ortín et al. (2007)	[187]				
**Sangiovese**	40.0	41.4	Medel-Maraboli et al. (2021)	[143]	109.0	361.9	Rinaldi et al. (2020)	[173]
	35.3	189.1	Rinaldi et al. (2020)	[173]	21.3	21.7	Tessarin et al. (2016)	[187]
	1.0	3.0	Tessarin et al. (2016)	[187]	14.7	18.8	Parpinello et al. (2015)	[188]
	1098.0	1110.0	Becatti et al. (2014)	[158]				
**Syrah**	37.6	53.4	Van Leeuw et al. (2014)	[138]	57.1	59.0	Gordillo et al. (2021a)	[191]
	38.9	40.1	Gordillo et al. (2021a)	[191]	132.7	161.9	Gordillo et al. (2021b)	[159]
	18.2	28.7	Gordillo et al. (2021b)	[159]	573.4	583.1	Nascimiento-Alves et al. (2022)	[160]
	43.3	48.7	Nascimiento-Alves et al. (2022)	[160]	46.9	146.5	Bian et al. (2026)	[161]
**Tannat**	6.9	8.8	Favre et al. (2014)	[162]	26.9	31.2	Favre et al. (2014)	[162]
	11.3	159.1	Vidal et al. (2018)	[174]	50.8	117.4	Vidal et al. (2018)	[174]
	9.4	10.0	Nascimiento-Alves et al. (2022)	[160]	278.5	719.9	Vidal et al. (2018)	[174]
	166.0	174.0	Boido et al. (2011)	[125]	319.7	329.1	Nascimiento-Alves et al. (2022)	[160]
					372.0	410.0	Boido et al. (2011)	[125]

Table S4. Mean concentration, dispersion (SD) and coefficient of variation (CV) of phenolic compounds in seeds, skins and wine

SEEDS					
**Compound**	**Cultivar**	**n (studies)**	**Mean estimate**	**SD**	**CV (%)**
Total phenols (mg/g)	Cabernet Sauvignon	4	39.39	27.86	70.7
Total phenols (mg/g)	Carménère	3	17.43	5.36	30.8
Total phenols (mg/g)	Grenache	2	101.28	51.37	50.7
Total phenols (mg/g)	Malbec	2	40.75	8.91	21.9
Total phenols (mg/g)	Merlot	4	233.24	327.49	140.4
Total phenols (mg/g)	Mourvèdre	1	54.65	n/a	n/a (n=1)
Total phenols (mg/g)	Sangiovese	3	29.01	17.6	60.7
Total phenols (mg/g)	Syrah	7	224.38	184.89	82.4
Total phenols (mg/g)	Tannat	2	110.79	76.66	69.2
Total tannins (mg/g)	Cabernet Sauvignon	3	25.6	33.04	129.1
Total tannins (mg/g)	Carménère	3	31.47	14.77	46.9
Total tannins (mg/g)	Grenache	3	66.55	61.37	92.2
Total tannins (mg/g)	Malbec	2	80.9	42.57	52.6
Total tannins (mg/g)	Merlot	4	119.34	211.83	177.5
Total tannins (mg/g)	Mourvèdre	2	65.48	88.35	134.9
Total tannins (mg/g)	Sangiovese	2	13.04	15.65	120
Total tannins (mg/g)	Syrah	4	96.75	41.27	42.7
Total tannins (mg/g)	Tannat	2	248.17	293.91	118.4
Gallic acid (mg/kg)	Cabernet Sauvignon	5	53.11	43.84	82.5
Gallic acid (mg/kg)	Carménère	3	75.43	47.8	63.4
Gallic acid (mg/kg)	Grenache	2	148.5	160.23	107.9
Gallic acid (mg/kg)	Malbec	2	74.32	21.11	28.4
Gallic acid (mg/kg)	Merlot	4	118.16	111.57	94.4
Gallic acid (mg/kg)	Mourvèdre	1	2.77	n/a	n/a (n=1)
Gallic acid (mg/kg)	Sangiovese	4	53.91	64.17	119
Gallic acid (mg/kg)	Syrah	4	419.3	458.38	109.3
Catechin (mg/kg)	Cabernet Sauvignon	5	773.11	685.63	88.7
Catechin (mg/kg)	Carménère	3	1968.77	282.54	14.4
Catechin (mg/kg)	Grenache	3	1651.8	1455.35	88.1
Catechin (mg/kg)	Malbec	2	3524.9	29.49	0.8
Catechin (mg/kg)	Merlot	5	2955.83	3312.7	112.1
Catechin (mg/kg)	Mourvèdre	2	789.54	1025.95	129.9
Catechin (mg/kg)	Sangiovese	4	1169.05	1260.61	107.8

Catechin (mg/kg)	Syrah	7	4878.76	9976.18	204.5
Catechin (mg/kg)	Tannat	2	1927.68	2509.78	130.2
Epicatechin (mg/kg)	Cabernet Sauvignon	5	553.31	459.48	83
Epicatechin (mg/kg)	Carménère	3	1321.42	506.08	38.3
Epicatechin (mg/kg)	Grenache	3	1340.54	1455.31	108.6
Epicatechin (mg/kg)	Malbec	2	2154.75	202.37	9.4
Epicatechin (mg/kg)	Merlot	4	2445.84	2956.02	120.9
Epicatechin (mg/kg)	Mourvèdre	2	346.45	415.14	119.8
Epicatechin (mg/kg)	Sangiovese	4	986.22	1096.19	111.1
Epicatechin (mg/kg)	Syrah	7	2646.14	5363.05	202.7
Epicatechin (mg/kg)	Tannat	2	1514.1	1871.15	123.6

		SKINS			
**Compound**	**Cultivar**	**n (studies)**	**Mean estimate**	**SD**	**CV (%)**
Total phenols (mg/g)	Cabernet Sauvignon	5	13.31	12.96	97.3
Total phenols (mg/g)	Carménère	3	5.22	4.81	92.3
Total phenols (mg/g)	Grenache	2	21.22	11.78	55.5
Total phenols (mg/g)	Malbec	2	14.12	6.19	43.8
Total phenols (mg/g)	Merlot	5	15.42	11.15	72.3
Total phenols (mg/g)	Mourvèdre	2	29.88	6.26	20.9
Total phenols (mg/g)	Sangiovese	3	18.11	11.53	63.7
Total phenols (mg/g)	Syrah	6	18.92	13.65	72.2
Total phenols (mg/g)	Tannat	2	19.08	1.74	9.1
Total anthocyanins (mg/g)	Cabernet Sauvignon	5	7.54	7.3	96.8
Total anthocyanins (mg/g)	Carménère	3	5.37	5.32	99.2
Total anthocyanins (mg/g)	Grenache	1	7.75	n/a	n/a (n=1)
Total anthocyanins (mg/g)	Malbec	2	9.22	8.17	88.5
Total anthocyanins (mg/g)	Merlot	7	11.02	9.27	84.2
Total anthocyanins (mg/g)	Mourvèdre	2	9.8	7.07	72.2
Total anthocyanins (mg/g)	Sangiovese	3	7.79	2.69	34.5
Total anthocyanins (mg/g)	Syrah	4	10.84	4.02	37.1
Total anthocyanins (mg/g)	Tannat	3	10.29	11.07	107.5
Total tannins (mg/g)	Cabernet Sauvignon	5	20.26	35.04	173
Total tannins (mg/g)	Carménère	3	7.7	2.36	30.6
Total tannins (mg/g)	Grenache	2	39.71	36.05	90.8
Total tannins (mg/g)	Malbec	1	22.25	n/a	n/a (n=1)
Total tannins (mg/g)	Merlot	4	21.91	38.77	176.9

Total tannins (mg/g)	Mourvèdre	2	34.96	48.14	137.7
Total tannins (mg/g)	Sangiovese	1	15.25	n/a	n/a (n=1)
Total tannins (mg/g)	Syrah	4	22.85	32.42	141.9
Catechin (mg/kg)	Cabernet Sauvignon	6	13.5	19.39	143.6
Catechin (mg/kg)	Carménère	3	2.07	0.56	27.2
Catechin (mg/kg)	Grenache	2	794.45	863.45	108.7
Catechin (mg/kg)	Malbec	1	5.35	n/a	n/a (n=1)
Catechin (mg/kg)	Merlot	6	31.58	28.31	89.6
Catechin (mg/kg)	Mourvèdre	1	1470	n/a	n/a (n=1)
Catechin (mg/kg)	Sangiovese	3	59.28	99.29	167.5
Catechin (mg/kg)	Syrah	8	229.62	358.49	156.1
Catechin (mg/kg)	Tannat	3	306.15	464.88	151.8
Epicatechin (mg/kg)	Cabernet Sauvignon	4	16.31	25.24	154.8
Epicatechin (mg/kg)	Grenache	2	179.68	29.45	16.4
Epicatechin (mg/kg)	Merlot	3	10.35	5.85	56.6
Epicatechin (mg/kg)	Mourvèdre	1	505	n/a	n/a (n=1)
Epicatechin (mg/kg)	Sangiovese	1	70.07	n/a	n/a (n=1)
Epicatechin (mg/kg)	Syrah	8	99.5	144.67	145.4
Epicatechin (mg/kg)	Tannat	2	5.74	0.48	8.4
**WINES**					
**Compound**	**Cultivar**	**n (studies)**	**Mean estimate**	**SD**	**CV (%)**
Total phenols (mg/L)	Cabernet Sauvignon	3	2113.89	534.38	25.3
Total phenols (mg/L)	Carménère	4	1839.38	506.8	27.6
Total phenols (mg/L)	Grenache	11	1700.63	378.55	22.3
Total phenols (mg/L)	Malbec	4	2188.7	1000.18	45.7
Total phenols (mg/L)	Merlot	3	1884	494.51	26.2
Total phenols (mg/L)	Mourvèdre	3	597.95	936.38	156.6
Total phenols (mg/L)	Sangiovese	4	1378.84	309.85	22.5
Total phenols (mg/L)	Syrah	7	1864.17	844.49	45.3
Total phenols (mg/L)	Tannat	6	1715.67	1120.06	65.3
Total anthocyanins (mg/L)	Cabernet Sauvignon	3	359.7	219.88	61.1
Total anthocyanins (mg/L)	Carménère	4	724.25	409.44	56.5
Total anthocyanins (mg/L)	Grenache	8	467.57	266.45	57
Total anthocyanins (mg/L)	Malbec	4	716.8	287.45	40.1
Total anthocyanins (mg/L)	Merlot	3	217.72	74	34
Total anthocyanins (mg/L)	Mourvèdre	5	393.57	119.66	30.4

Total anthocyanins (mg/L)	Sangiovese	4	319.78	199.23	62.3
Total anthocyanins (mg/L)	Syrah	5	258.09	264.32	102.4
Total anthocyanins (mg/L)	Tannat	8	512.29	417.17	81.4
Total tannins (mg/L)	Cabernet Sauvignon	3	1666.04	1031.1	61.9
Total tannins (mg/L)	Carménère	3	1029.17	1233.6	119.9
Total tannins (mg/L)	Grenache	6	1485	562.8	37.9
Total tannins (mg/L)	Malbec	4	2325.87	1692.92	72.8
Total tannins (mg/L)	Merlot	3	1562.82	1029.28	65.9
Total tannins (mg/L)	Mourvèdre	3	1055.73	560.78	53.1
Total tannins (mg/L)	Sangiovese	3	1771.61	1785.08	100.8
Total tannins (mg/L)	Syrah	4	726.96	297.44	40.9
Total tannins (mg/L)	Tannat	4	3043.12	1708.99	56.2
Catechin (mg/L)	Cabernet Sauvignon	4	49.87	27.05	54.2
Catechin (mg/L)	Carménère	6	16.62	8.18	49.2
Catechin (mg/L)	Grenache	3	52.62	48.6	92.4
Catechin (mg/L)	Malbec	4	15.44	14.3	92.6
Catechin (mg/L)	Merlot	5	47.17	7.47	15.8
Catechin (mg/L)	Mourvèdre	3	19.42	12.2	62.8
Catechin (mg/L)	Sangiovese	4	35.64	43.16	121.1
Catechin (mg/L)	Syrah	7	56.64	78.36	138.3
Catechin (mg/L)	Tannat	4	27.91	25.65	91.9
Epicatechin (mg/L)	Cabernet Sauvignon	4	28.06	26.15	93.2
Epicatechin (mg/L)	Carménère	6	7.84	5.52	70.4
Epicatechin (mg/L)	Grenache	3	14.43	11.94	82.7
Epicatechin (mg/L)	Malbec	4	12.74	9.39	73.7
Epicatechin (mg/L)	Merlot	5	43.57	18.38	42.2
Epicatechin (mg/L)	Mourvèdre	3	7.22	7.48	103.7
Epicatechin (mg/L)	Sangiovese	4	11.3	7.54	66.7
Epicatechin (mg/L)	Syrah	7	31.84	39.37	123.7
Epicatechin (mg/L)	Tannat	3	39.56	28.96	73.2
Malvidin-3-glucoside (mg/L)	Cabernet Sauvignon	6	120.13	73.87	61.5
Malvidin-3-glucoside (mg/L)	Carménère	3	115.52	133.31	115.4
Malvidin-3-glucoside (mg/L)	Grenache	4	262.69	203.98	77.6
Malvidin-3-glucoside (mg/L)	Malbec	4	150.3	119.6	79.6
Malvidin-3-glucoside (mg/L)	Merlot	6	70.61	31.05	44
Malvidin-3-glucoside (mg/L)	Mourvèdre	4	106	58.38	55.1
Malvidin-3-glucoside (mg/L)	Sangiovese	4	32.78	27.76	84.7

Malvidin-3-glucoside (mg/L)	Syrah	8	62.59	23.71	37.9
Malvidin-3-glucoside (mg/L)	Tannat	7	178.62	167.87	94
Flavonols (mg/L)	Cabernet Sauvignon	3	63.42	58.71	92.6
Flavonols (mg/L)	Carménère	3	58.78	37.81	64.3
Flavonols (mg/L)	Grenache	3	21.05	19.83	94.2
Flavonols (mg/L)	Malbec	4	43.72	36.33	83.1
Flavonols (mg/L)	Merlot	3	39.89	19.16	48
Flavonols (mg/L)	Mourvèdre	4	44.59	32.65	73.2
Flavonols (mg/L)	Sangiovese	4	314.73	528.16	167.8
Flavonols (mg/L)	Syrah	4	38.61	10.52	27.2
Flavonols (mg/L)	Tannat	4	68.19	76.85	112.7
Phenolic acid (mg/L)	Cabernet Sauvignon	3	63.41	54.5	86
Phenolic acid (mg/L)	Carménère	2	37.35	22.06	59.1
Phenolic acid (mg/L)	Grenache	3	49.08	60.98	124.2
Phenolic acid (mg/L)	Malbec	3	33.96	16.79	49.4
Phenolic acid (mg/L)	Merlot	3	176.76	176.99	100.1
Phenolic acid (mg/L)	Mourvèdre	3	58.2	34.89	59.9
Phenolic acid (mg/L)	Sangiovese	3	91.22	124.93	136.9
Phenolic acid (mg/L)	Syrah	4	220.08	241.56	109.8
Phenolic acid (mg/L)	Tannat	5	265.55	201.65	75.9

Mean estimate and SD calculated from the midpoint [(min+max)/2] of each individual study's reported range, aggregated across all studies available for each cultivar-compound combination. CV = SD/Mean x 100. n indicates the number of independent studies contributing to each estimate; estimates based on n<=2 should be interpreted with caution.
